# Thrombectomy for Ischemic Stroke Beyond 24 Hours: A Meta-Analysis

**DOI:** 10.3390/life15040556

**Published:** 2025-03-28

**Authors:** Hao-Tse Chiu, Po-Huang Chen, Yen-Yue Lin, Li-Yu Yang, Cho-Hao Lee, Che-Yu Guan, Hong-Jie Jhou

**Affiliations:** 1 Department of Emergency Medicine, Taoyuan Armed Forces General Hospital, National Defense Medical Center, No. 168, Zhongxing Rd., Longtan Dist., Taoyuan 325208, Taiwan; haotsechiu@gmail.com (H.-T.C.); yyline.tw@yahoo.com.tw (Y.-Y.L.); 2 Department of Emergency Medicine, Tri-Service General Hospital, National Defense Medical Center, Taipei 11490, Taiwan; 3 Division of Hematology and Oncology Medicine, Department of Internal Medicine, Tri-Service General Hospital, National Defense Medical Center, Taipei 11490, Taiwan; chenpohuang@hotmail.com (P.-H.C.); drleechohao@gmail.com (C.-H.L.); 4 School of Medicine, Kaohsiung Medical University, Kaohsiung 807378, Taiwan; vicky102433@gmail.com; 5 Department of Neurology, Chang Bing Show Chwan Memorial Hospital, Changhua 50544, Taiwan; 6 Department of Neurology, Changhua Christian Hospital, 135 Nanhsiao Street, Changhua 50006, Taiwan

**Keywords:** acute ischemic stroke, endovascular thrombectomy, large vessel occlusion, low NIHSS score, meta-analysis

## Abstract

Background: The DEFUSE-3 and DAWN studies established the benefits of endovascular therapy for patients with stroke with large vessel occlusion in a 6–24 h time window. However, the effectiveness of endovascular therapy performed beyond 24 h remains uncertain. The purpose of this meta-analysis is to evaluate the difference in prognosis between thrombectomies performed beyond 24 h and within 24 h from ischemic stroke onset. Methods: A systematic review was conducted using the PubMed, Cochrane, and Embase databases from database inception until 1 February 2024. Odds ratios with 95% confidence intervals were calculated. Results: This study included seven cohort articles involving 6137 participants who received endovascular therapy, with 395 patients in the beyond 24 h group and the remainder in the within 24 h group. The results for functional independence, successful reperfusion, any intracranial hemorrhage, symptomatic intracranial hemorrhage, and 90-day mortality rates were similar between the two groups, with odds ratios of 1.06 (95% confidence interval: 0.51–2.19), 1.03 (0.72–1.48), 0.88 (0.64–1.21), 0.76 (0.41–1.40), and 1.32 (0.55–3.19), respectively. Furthermore, all trial sequential analysis results were inconclusive. Conclusions: Functional independence, successful reperfusion, mortality, and intracranial hemorrhage rates did not significantly differ between endovascular therapies performed beyond and within 24 h from ischemic stroke onset. Therefore, endovascular therapy may be considered for patients experiencing ischemic stroke for more than 24 h. However, randomized controlled trials and more cohort studies are needed to confirm these conclusions.

## 1. Introduction

Approximately half of patients with stroke with large vessel occlusion (LVO) experience slow disease progression, developing small core infarcts within 6–24 h [1]. Thus, the DEFUSE-3 and DAWN trials have significantly extended the time window of thrombectomy to 6–24 h [2,3], marking a pivotal advancement in stroke treatment. Further reinforcing this extension, the AURORA study reported that endovascular therapy (EVT) is effective for patients exhibiting signs of reversible cerebral ischemia within 6–24 h [4].

The penumbra reportedly persists for up to 48 h after stroke onset [4,5]. Without rebuilding cerebral blood flow or sufficient collateral circulation, the penumbral tissue may progress to ischemic infarction. The MR CLEAN-LATE study found that EVT may improve neurological outcomes in patients with stroke in the late window who had maintained collateral circulation [6]. A post hoc analysis of the DEFUSE-3 trials revealed poorer outcomes for stroke patients beyond 24 h receiving medical treatment alone [7] but a likely favorable prognosis for those undergoing reperfusion therapy. Likewise, the Select Late Study inferred that even in large vessel strokes beyond 24 h [8], the rate of functional independence was higher in patients undergoing EVT than in those receiving medical treatment alone. However, the effectiveness of EVT beyond 24 h still has no high-quality evidence.

Thus, this meta-analysis aimed to evaluate the effectiveness of thrombectomy beyond 24 h for patients with stroke with LVO.

## 2. Materials and Methods

This systematic review and meta-analysis was rigorously conducted following a pre-registered protocol (CRD42024513378). Our report adheres to the Preferred Reporting Items for Systematic Reviews and Meta-Analyses guidelines (PRISMA) (Supplementary Information S1) [9]. Given that meta-analyses synthesize data from existing published studies, this study did not necessitate ethical approval. It should provide a concise and precise description of the experimental results, their interpretation, and the experimental conclusions that can be drawn.

### 2.1. Search Design

We extensively searched for relevant studies from three major databases, namely Medline, Embase, and the Cochrane Central Register of Controlled Trials (Supplementary Information S2), beginning from the inception of each database to 1 February 2024, with no language limitations. Our search strategy incorporated a carefully crafted set of keywords as follows: “Embolectomy OR Thrombectomy OR Endovascular”, “Stroke OR CVA OR Cerebrovascular Accident”, and “Beyond 24 Hours OR Very Late Window OR Very Late Time OR Late Time”. Additionally, we explored supplementary sources such as conference abstracts, trial registries (e.g., ClinicalTrials.gov), and presentations at conferences to access unpublished data.

### 2.2. Study Selection

Studies that assessed the effectiveness of thrombectomy in adult patients (aged ≥18 years) with LVO stroke and compared outcomes in very late windows (beyond 24 h) with those within 24 h were included. To ensure data integrity and avoid redundancy (e.g., instances of participant sample overlap), we preferred the most recent studies. We systematically excluded the following study types: studies without a control arm, studies with unclear outcome measurement, studies not directly relevant to our population, case reports, and conference abstracts.

### 2.3. Outcome Measurements

The outcomes were defined as follows: a good functional outcome characterized by a modified Rankin Scale (mRS) score of 0–2 at 90 days; a successful reperfusion denoted by a modified Thrombolysis in Cerebral Infarction (mTICI) scale score of 2b/3; the rate of any intracranial hemorrhage (aICH); the rate of symptomatic intracranial hemorrhage (sICH); and mortality at 90 days.

### 2.4. Data Extraction

Titles and abstracts, which were retrieved from our comprehensive search, were initially screened by two authors (H.J.J. and H.T.C.) independently to sift out articles not pertinent to our study objectives. Subsequently, full-text articles that met the inclusion criteria were rigorously evaluated by at least two independent reviewers. During study selection, any disputes were amicably resolved through discussions, or, when necessary, by consulting a third author (P.H.C.) for an impartial decision. Despite suggestions favoring a per-protocol analysis for potentially larger effect sizes, our study strictly adhered to an intention-to-treat approach to maintain methodological integrity.

### 2.5. Risk of Bias Assessment

The included cohort studies’ methodological quality was independently evaluated by two authors (H.J.J. and H.T.C.) using the Newcastle–Ottawa Scale (NOS) (Supplementary Information S3) [10]. Any evaluative discrepancies were resolved through discussions or, if necessary, consultation by a third author (P.H.C.). The NOS assesses the quality of nonrandomized studies, particularly cohort studies. Studies were judged on these parameters and assigned a score. Total scores of 0–3, 4–6, and 7–9 points indicate low, intermediate, and high quality, respectively. This comprehensive assessment included considerations of the exposed cohort’s representativeness, selection of the nonexposed cohort, ascertainment of exposure, demonstration that the outcome of interest was not present at the start of the study, comparability of cohorts according to the design or analysis, assessment of the outcome, sufficient follow-up length for outcomes to occur, and adequacy of cohort follow-up.

### 2.6. Handling of Missing Data

Proactive measures were taken to mitigate the impact of missing data. One measure was to contact the original trial authors to acquire any missing data. When such measures were unsuccessful in obtaining the missing data, the analysis proceeded using the available data. We consciously refrained from employing any imputation techniques used for estimating missing data because such techniques might introduce potential biases to our analysis results.

### 2.7. Statistical Analysis

Our statistical analysis was rigorously conducted using both fixed-effect and random-effects models as outlined in the *Cochrane Handbook for Systematic Reviews of Interventions* [11]. In the fixed-effect model, homogeneity is assumed among effect sizes, while in the random-effects model, potential variability is considered across studies. In our approach, we used the inverse variance method and applied the DerSimonian–Laird estimator for calculating tau-squared [12]. Additionally, the confidence interval (CI) for tau-squared and tau was determined using the Jackson method [13]. For categorical outcomes, we calculated odds ratios (ORs) with 95% CIs. We also employed the I2statistic and the Cochrane Q test, with I2 values over 50% and a Cochrane Q *p*-value less than 0.10 indicating substantial heterogeneity [14]. Small-study effects were examined using the regression-based Egger’s test, and publication bias was assessed with funnel plots for each outcome (Supplementary Information S4) [15]. All data were managed and analyzed using the “metafor” and “meta” packages in R software (version 4.3.1, R Foundation for Statistical Computing, Vienna, Austria). A two-tailed *p*-value less than 0.05 was considered statistically significant.

### 2.8. Trial Sequential Analysis

We employed a TSA to augment our meta-analysis, especially when the available data were inadequate for conclusive interpretations. TSA integrates the estimated required information size and implements a modified significance threshold, thereby mitigating premature overestimations. This method ensures the reliability and validity of our findings, countering potential limitations in the existing data [16]. We adopted a TSA model with a 5% two-sided α (0.05) type 1 error rate and 80% statistical power, in line with the O’Brien–Fleming alpha-spending function [17]. For dichotomous outcomes, event rates in the treatment and control groups generally indicate the proportion of patients experiencing the outcome of interest in each group. Relative risk reduction is then calculated according to these rates. For continuous outcomes, treatment effects were quantified using the standardized mean difference, which can be derived from the overall mean difference and variance of the outcome across all included trials. The TSA was executed using TSA software (version 0.9.5.10 Beta; Copenhagen Trial Unit, Copenhagen, Denmark).

### 2.9. Quality Assessment

The quality of evidence for each outcome was rigorously evaluated using the Grading of Recommendations Assessment, Development, and Evaluation (GRADE) approach, facilitated by GRADEpro software (version 20; McMaster University, Hamilton, ON, Canada, 2014) (Supplementary Information S5) [18]. This comprehensive assessment encompassed multiple factors, including the bias risk, inconsistency, indirectness, imprecision, and publication bias. On the basis of these criteria, outcomes were systematically categorized as having high, moderate, low, or very low certainty.

### 2.10. Time to Groin Puncture and Outcome Measures

We analyzed the relationship between the time from last known well (TLKW) to groin puncture (median hours) and various clinical outcomes in patients undergoing mechanical thrombectomy beyond 24 h. Scatter plots were created to visually illustrate the association between time and pooled odds ratios (ORs), with a linear regression line fitted to show the trend. A reference line at OR = 1 was added to indicate the threshold for a neutral effect, and each study was labeled for easy identification.

## 3. Results

### 3.1. Search Strategy

**Figure 1:**
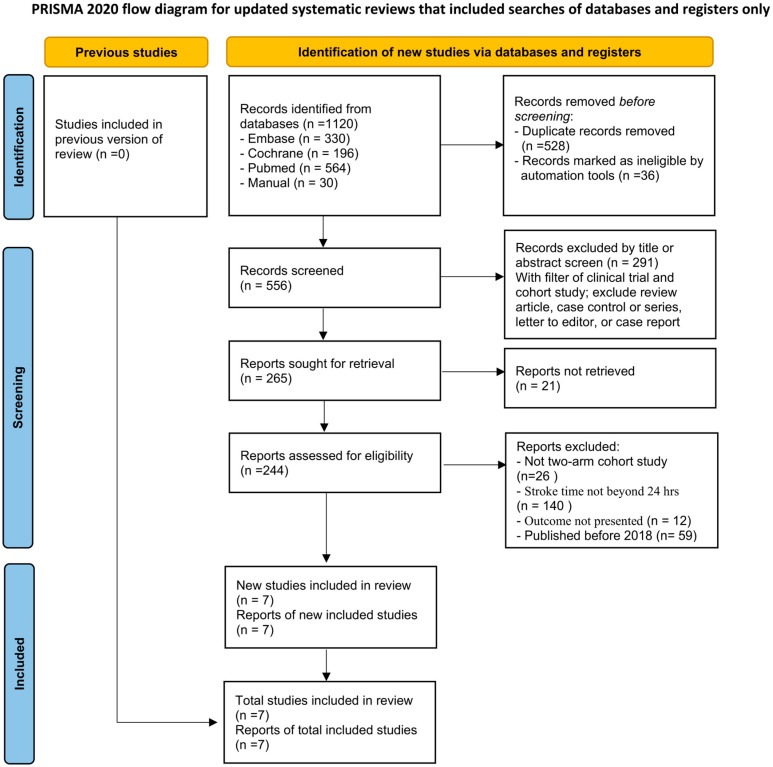
Flow diagram of the identification process for eligible studies.

We found 1120 studies, of which 528 records were excluded because of duplicates (Figure 1). After reviewing the titles and abstracts and assessing the full text, we excluded 585 articles, leaving 7 articles that met our eligibility criteria [19,20,21,22,23,24,25]. These trials included 6137 participants, with 395 patients undergoing EVT beyond 24 h and the remainder receiving EVT within 24 h (Table 1).

Four studies were high-quality studies [19,20,21,24] and the other studies were rated as intermediate-quality studies (Supplementary Information S3) [22,23,25]. The median age of the participants ranged from 60.0 to 75.5 years. The median baseline mRS score ranged from 0 to 2. The median baseline National Institutes of Health Stroke Scale (NIHSS) score ranged from 10 to 18. The study participants included individuals from the United States, Europe, the United Kingdom, and Asia. The characteristics of all included trials are outlined in Supplementary Information S7.

### 3.2. Primary Outcomes

#### Good Functional Outcomes

**Figure 2:**
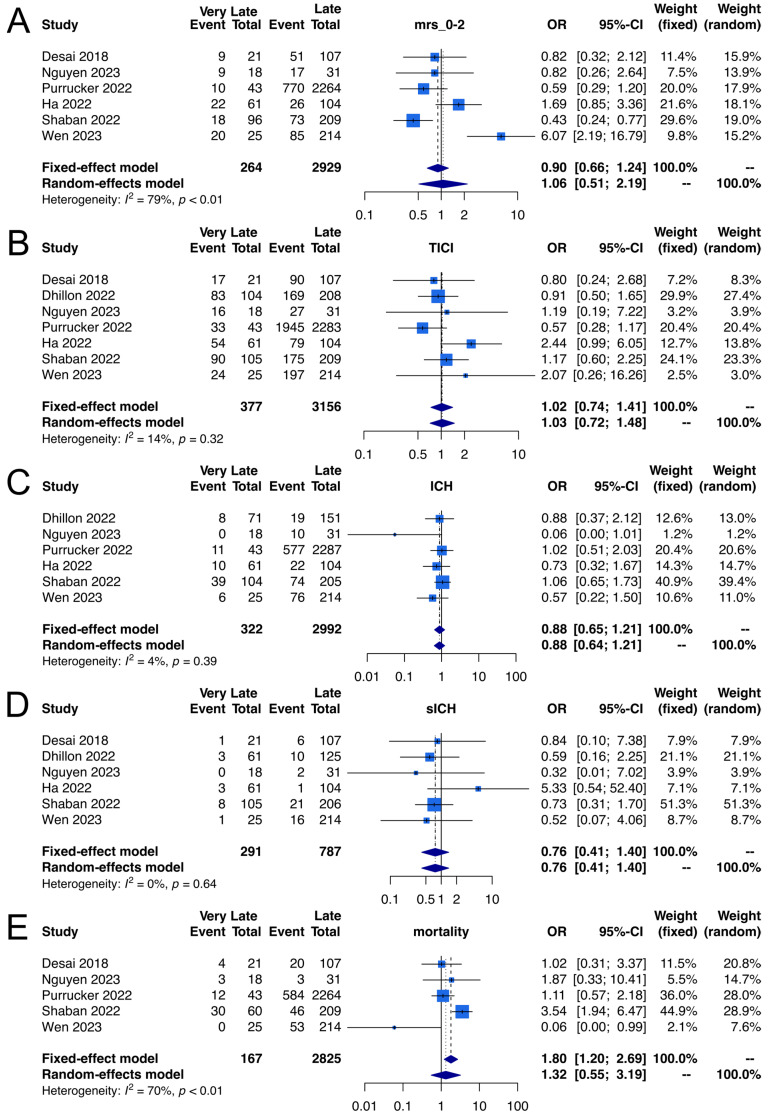
Meta-analysis of outcomes regarding the 90-day mRS score of 0–2 (**A**) [19,21,22,23,24,25], mTICI scale score of 2b/3 (**B**) [19,20,21,22,23,24,25], aICH (**C**) [20,21,22,23,24,25], sICH (**D**) [19,20,21,23,24,25], and 90-day all-cause mortality (**E**) [19,21,22,24,25]. mRS, modified Rankin Scale; mTICI, modified Thrombolysis in Cerebral Infarction; aICH, any intracranial hemorrhage; sICH, symptomatic intracranial hemorrhage.

**Figure 3:**
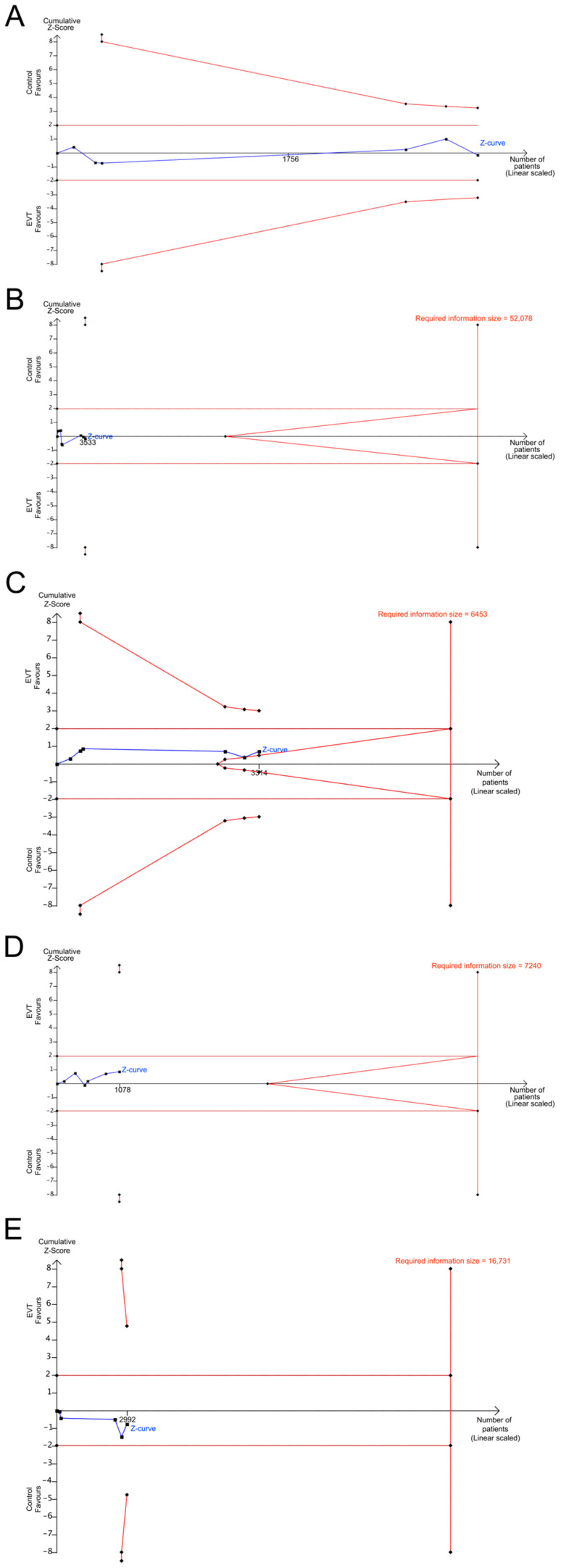
Trial sequential analysis (TSA) of outcomes regarding the 90-day mRS score of 0–2 (**A**), mTICI scale score of 2b/3 (**B**), aICH (**C**), sICH (**D**), and 90-day all-cause mortality (**E**). The *X*-axis represents the total number of patients included in the analysis, and the *Y*-axis indicates the cumulative Z score. Horizontal dark red lines mark the conventional boundaries used to determine statistical significance. The sloping red lines at the top and bottom left-hand corners, also known as trial sequential boundaries, signify the thresholds required for statistical significance within the context of TSA. Red diagonal lines within the horizontal brown lines delineate the futility boundaries, suggesting areas where continuing the trial is unlikely to yield significant results. The full vertical red line indicates the required information size, the total amount of data needed to reach a conclusive result. The solid blue line represents the cumulative z-curve, showing the progression of the evidence of the statistical test over the course of the study.

Good functional outcomes were examined in six studies with a total of 3193 participants [19,21,22,23,24,25]. The outcomes were comparable between patients who underwent EVT beyond 24 h and those within 24 h (33.3% vs. 34.9%; OR, 1.06; 95% CI, 0.51–2.19; I^2^ = 79%; *p*-value for heterogeneity <0.01; Figure 2A). However, TSA findings indicated inconclusive evidence. The TSA graph for good functional outcomes showed that the z-curve neither crossed the required information size nor any monitoring boundaries, suggesting that further data are needed to confirm these results (Figure 3A).

### 3.3. Secondary Outcomes

#### Successful Reperfusion

Data regarding successful reperfusion were provided by seven trials encompassing 3533 participants [19,20,21,22,23,24,25]. The outcomes were similar between patients who underwent EVT beyond 24 h and those within 24 h (84.1% vs. 85.0%; OR, 1.03; 95% CI, 0.72–1.48; I^2^ = 14%, *p*-value for heterogeneity = 0.32; Figure 2B). However, TSA results were inconclusive; in the graph, the z-curve neither crossed the required information size nor any monitoring boundaries, suggesting a need for further data to confirm these results (Figure 3B).

### 3.4. Safety Outcomes

#### 3.4.1. Any ICH

Six trials encompassing 3314 participants provided data regarding aICH [20,21,22,23,24,25]. The outcomes were similar between the two abovementioned patient groups (23.0% vs. 26.0%; OR, 0.88; 95% CI, 0.64–1.21; I2 = 4%, *p*-value for heterogeneity = 0.39; Figure 2C). However, the TSA findings were inconclusive; the graph showed that the z-curve neither crossed the required information size nor any monitoring boundaries, suggesting that further data are needed to confirm these results (Figure 3C).

#### 3.4.2. Symptomatic ICH

Data regarding sICH were provided by six trials encompassing 1078 participants [19,20,21,23,24,25]. The outcomes were also similar between the two patient groups (5.5% vs. 7.1%; OR, 0.76; 95% CI, 0.41–1.40; I2 = 0%, *p*-value for heterogeneity = 0.64; Figure 2D), with inconclusive TSA results. The TSA graph for sICH showed that the z-curve neither crossed the required information size nor any monitoring boundaries; hence, further data are required to confirm these results (Figure 3D).

#### 3.4.3. Mortality at 90 Days

Five trials including 2992 participants provided data regarding mortality at 90 days [19,21,22,24,25]. The outcomes were comparable between the two patient groups (29.3% vs. 25.0%; OR, 1.32; 95% CI, 0.55–3.19; I2 = 70%, *p*-value for heterogeneity <0.01; Figure 2E), and the TSA findings indicated inconclusive evidence; in the graph, the z-curve neither crossed the required information size nor any monitoring boundaries, suggesting a need for further data to confirm these results (Figure 3E).

### 3.5. Publication Bias and Quality Assessment

In our analysis, we generally did not detect any publication bias through funnel plots and the Egger’s test, with one notable exception. Specifically, the Egger’s test result for the aICH outcome was significant (*p* = 0.018); thus, publication bias might be possible. However, these findings must be interpreted with caution. The limited number of studies included in our meta-analysis for this particular outcome could potentially influence the assessment of publication bias. This small sample size may lead to bias overestimation or underestimation, thereby affecting the reliability of the Egger’s test in this context. Consequently, while the Egger’s test indicates potential publication bias for the ICH outcome, a careful and nuanced interpretation of these results is necessary because of the limited data available. Supplementary Information S6 summarizes the GRADE assessments [26]. The certainty of evidence regarding all outcomes was deemed very low.

### 3.6. Association Between Time to Groin Puncture and Clinical Outcomes

The scatter plots revealed distinct trends in the relationship between time last known well to groin puncture time and clinical outcomes. All outcomes except mortality showed a positive correlation with increasing time, while the odds ratio for any ICH was less than 1. For mortality at 90 days, limited data precluded the identification of a definitive trend (Supplementary Information S6).

## 4. Discussion

This meta-analysis showed no significant differences in all outcomes between patients undergoing EVT beyond 24 h versus within 24 h from the onset of acute ischemic stroke. Therefore, thrombectomy for patients beyond 24 h may be a reasonable and feasible procedure. Although the TSA indicates that EVT remains effective for patients beyond 24 h, conclusive evidence remains elusive because of the limited number of cases. To our knowledge, this study is the first to comprehensively analyze the effectiveness of EVT beyond 24 h for patients with LVO.

In the DEFUSE 3 study’s post hoc analysis, one-fifth of the patients in the very late window who did not receive EVT exhibited a persistent mismatch for at least an additional 24 h [27]. Patients with robust collateral circulation may maintain the penumbra and limit ischemic core size. Nevertheless, without thrombectomy, collateral failure may occur, enlarging the ischemic core and worsening patients’ prognosis [28]. Furthermore, patients undergoing EVT beyond 24 h have better outcomes than those treated with medical management alone [8,29]. In our results, approximately one-third of patients in both groups achieved functional independence. Factors such as age, initial NIHSS score, and anterior and posterior circulation stroke also influence the recovery of functional independence. In the studies by Ha and Wen [23,25], patients undergoing EVT beyond 24 h were younger and had lower initial NIHSS scores; subsequently, they achieved better outcomes in the beyond 24 h group, thus introducing higher heterogeneity in functional independence at 90 days. Shaban found that for anterior circulation LVO beyond 24 h, functional independence at 90 days was significantly worse than those within 6–24 h [24]. No difference was observed in posterior circulation LVO outcomes. Therefore, subsequent research should focus on identifying patients for whom EVT beyond 24 h may be more effective.

Moreover, the successful reperfusion rates between each group were comparable. Thus, achieving reperfusion in cerebral vasculature even beyond 24 h from stroke onset may be technically feasible. Ha et al. [23] found that irrespective of the time window, early neurological deterioration (END) was solely associated with complete reperfusion failure. The current causes of END remain uncertain. Identifying triggers for END can reduce the risk of disability and death [30]. However, successful reperfusion did not significantly correlate with early neurological improvement [31,32]. Additionally, Sarraj et al. [33] noted that larger volumes of critically hypoperfused tissue shown on imaging were more likely to culminate in END. Furthermore, despite mechanical thrombectomy being conducted beyond 24 h, we found no significant rise in aICH, sICH, and 90-day mortality rates.

In the reviewed studies, physicians performed EVT based on individual cases [20,22,23,24,25] or perfusion imaging [19,21], which may have introduced selection bias. These considerations included a relatively small ischemic core on imaging and the proportion of salvageable penumbra documented on perfusion imaging. Additionally, the extent of collateral circulation may have also impacted infarct size progression. Therefore, alongside the time window, the aforementioned factors should be comprehensively considered to select appropriate patients for the treatment.

Recently, three meta-analyses have investigated endovascular thrombectomy (EVT) beyond 24 h after stroke onset. Studies by Rodriguez-Calienes [34] and Kobeissi et al. [35] demonstrated favorable outcomes of EVT beyond 24 h, suggesting its feasibility. However, both studies were single-arm designs. In contrast, Shakir et al. [36] compared the outcomes of EVT beyond 24 h with those within 24 h using a double-arm design. Nevertheless, they included only four studies and did not perform TSA to address type I and type II errors. Detailed comparisons of these studies are presented in Table 2.

This study has several strengths. It is an updated meta-analysis exploring the outcomes of EVT within and beyond 24 h. The TSA was employed to reduce bias and provide more precise effect size estimates. Furthermore, the GRADE was used to assess the certainty of the evidence, which could be valuable for physicians and patients in making informed decisions. Recent randomized control trials demonstrate endovascular treatment efficacy in patients with large ischemic cores, supported by consistent meta-analytic findings [37]. While infarct cores may expand beyond 24 h post-onset, these positive outcomes suggest broader thrombectomy criteria, indicating greater tissue salvageability than previously recognized. Our study has several limitations. First, the lack of consensus on mechanical thrombectomy for strokes beyond 24 h and the nature of retrospective study design both contribute to the possibility of selection bias. Second, while this study mainly included patients with stroke affecting the anterior circulation, posterior circulation cases were scarce. Hence, the findings are not fully representative or consistent, warranting cautious interpretation. Third, the TSA findings indicate that the current sample size may be inadequate to derive conclusive results. Consequently, additional research is necessary in the future. One example of such research includes the ongoing LATE-MT (Large Artery Occlusion Treated in Extended Time With Mechanical Thrombectomy) trial, which is enrolling patients with LVO undergoing EVT after 24 h (http://clinicaltrials.gov; Unique identifiers: NCT05326932).

## 5. Conclusions

EVT beyond 24 h from stroke onset yields comparable functional outcomes without increasing the risk of ICH. This indicates that the time window may not necessarily represent a biological limit for the salvageability of the penumbra. Future randomized controlled trials are needed to confirm the effectiveness of thrombectomy for patients with stroke with LVO beyond 24 h and to identify suitable candidates, thereby minimizing the occurrence of complications.

## Figures and Tables

**Table 1:** Characteristics of included studies.

Author, Year	Study Design	Country	No. of Patients	Age (year)	Male (%)	NIHSS	ASPECTS	HTN (%)	DM (%)	AF (%)	Previous Stroke (%)	tPA (%)	Occlusion Site, ICA (%)	Occlusion Site, MCA (%)	Occlusion Site, Other (%)	TLKW to Groin Puncture Hour (Median)	Overall RoB
Desai 2018 [19]	cohort	U.S.A	>24: 21	65.2 ± 11.1	38.1	18.2 ± 5.9	-	81	33	9.5	-	-	52.0	48.0	0-	48 (30–72)	Int.
			6–24: 107	69.4 ± 14.1	39.2	17.4 ± 5.1	-	78	24	40	-	-	21.0	78.0-	0-	12.8 (10.6–16.7)	
Dhillon 2023 [20]	cohort	U.K.	>24: 104	-	57.7	12.7 ± 7.4	-	48.0	10.5	17.3	9.6	19.2	-	-	-	33.5 ± 7.4	Int.
			6–24: 1046	-	53.0	15.2 ± 7.7	-	46.7	13.3	19.3	13.9	31.5	-	-	-	10.5 ± 4.2	
Nguyen2023 [21]	cohort	Vietnam	>24: 20	60.0 (54.0–68.0)	85.0	12(10–18)	8	85.0	20.0	10.0	-	0.0	55.0	30.0	15.0	27.2 (25.7–30.9)	High
			6–24: 146	63.5 (56.2–70.0)	69.2	15(11–18)	8	86.3	17.8	18.5	-	6.45	32.2	60.3	7.5	14.3 (11.9–18.9)	
Purrucker 2022 [22]	cohort	German	>24: 43	75.5 ± 10.1	46.5	13(8–21)	9	74.4	27.9	30.2	11.6	7.0	4.7	37.2	58.1	-	Int.
			6–24: 2304	73.9 ± 12.7	48.2	15(9–21)	9	76.0	23.4	46.7	21.6	49.0	4.9	58.2	36.9	-	
Ha 2022 [23]	cohort	South Korea	>24: 61	65.0 ± 14.0	70.5	10.0 ± 6.0	-	78.7	24.6	16.4	28.1	4.1	-	-	-	80.8 (43.8–194.2)	Int.
			6–24: 104	71.0 ± 12.0	60.6	13.0 ± 7.0	-	52.9	26.9	49.0	16.9	23.7	-	-	-	10 (8.0–14.9)	
Shaban 2022 [24]	cohort	U.S.A	>24: 91	67.0 ± 13.3	55.4	14.0 ± 9.0	8	78.5	35.5	17.6	28.1	4.1	-	-	-	-	High
			6–24: 214	68.1 ± 15.0	49.2	15.0 ± 7.0	8	73.6	29.1	32.7	16.9	23.7	-	-	-	-	
Wen 2023 [25]	cohort	China	>24: 25	62.8 ± 2.0	88	12.8 ± 0.6	7	52.0	24.0	16.0	-	20	16.0	52.0	32.0	-	Int.
			6–24: 214	67.8 ± 0.8	58.9	17.2 ± 0.4	7	56.1	11.7	47.7	-	46.3	9.8	64.5	25.7	-	

NIHSS: National Institutes of Health Stroke Scale; ASPECTS: The Alberta Stroke Program Early CT score; HTN: hypertension; DM: diabetes mellitus; tPA: tissue plasminogen activator; ICA: internal carotid artery; MCA: middle cerebral artery; TLKW: time last known well; RoB: risk of bias; Int: intermediate.

**Table 2:** Comparison with other previous meta-analyses.

Author, Year	Rodriguez-Calienes, 2024 [34]	Kobeissi, 2023 [35]	Shakir, 2024 [36]	Our Meta-Analysis, 2025
No. of studies	12	7	4	7
No. of individuals	517	569	312	6137
Search strategy until	April 2023	December 2022	2024	Feb 2024
Study design	Single-arm	Single-arm	double-arm	double-arm
The good function outcome at 3 months; OR; 95% CI	40 (31 to 49) *	32.0 (24.7–40.2) *	0.85 (0.34–2.09)	1.06 (0.51–2.09)
Successful reperfusion; OR; 95% CI	83 (80 to 85) *	81.9 (78.5–84.9) *	Not reported	1.03 (0.72–1.48)
Any ICH; OR; 95% CI	25 (18 to 35) *	Not reported	0.98 (0.76–1.26)	0.88 (0.64–1.21)
sICH; OR; 95% CI	7 (5 to 9) *	6.80 (4.3–10.7) *	0.85 (0.44–1.64)	0.76 (0.41–1.40)
Mortality at 3 months; OR; 95% CI	28 (24 to 33) *	27.2 (22.9–31.9) *	1.08 (0.73–1.61)	1.32 (0.55–3.19)
Trial sequential analysis	Not applied	Not applied	Not applied	Applied
Evidence of effect	Not applied	Not applied	Not applied	Inconclusive
GRADE	Low: favorable functional outcome, successful reperfusion, sICH, 90-day mortality Very low: ICH	Not applied	Not applied	Very low

CI = confidence interval; GRADE = Grading of Recommendations Assessment, Development, and Evaluation; OR = odds ratio; sICH = symptomatic intracerebral hemorrhage. * Proportional meta-analysis data.

## Data Availability

All data relevant to the study are included in the article or uploaded as Supplementary Information.

## Supplementary Materials

The following supporting information can be downloaded at https://www.mdpi.com/article/10.3390/life15040556/s1.
