# Supplementary material for: Thrombectomy for Ischemic Stroke Beyond 24 Hours: A Meta-Analysis
Source: Life (Basel). 2025 Mar 28;15(4):556. doi: 10.3390/life15040556 (PMC12028478; doi:10.3390/life15040556)
Supplement: Supplementary file 1 [file life-15-00556-s001.zip › life-3461150-supplementary.pdf]

Supplementary Information

Title

Hao-Tse Chiu, MD[1,2]; Po-Huang Chen, MD[3]; Yen-Yue Lin, MD[1,2]; Li-Yu Yang, MD [4,5]; Cho-Hao Lee, MD[3]; Chung-Cheng Kao, MD [1+]; Che-Yu Guan[1,2+]; Hong-Jie Jhou, MD[4,6]*

Contents

Supplementary Information 1. PRIMSA Checklist
Supplementary Information 2. Search strategy
Supplementary Information 3. Assessment of risk of bias
Supplementary Information 4. Contour-enhanced meta-analysis funnel plots and Egger's test
Supplementary Information 5. The Grading of Recommendations, Assessment, Development and Evaluations
Supplementary Information 6. Scatter Plot of the Association Between Time from Last Known Well (TLKW) to Groin Puncture (Median Hours) and Pooled Odds Ratios (ORs)
Supplementary Information 7. Detail of study characteristics

Information 1 PRIMSA checklist

Section/topic	Item No	Checklist item	Reported on page No
**Title**			
Title	1	Identify the report as a systematic review, meta-analysis, or both	P1
**Abstract**			
Structured summary	2	Provide a structured summary including, as applicable, background, objectives, data sources, study eligibility criteria, participants, interventions, study appraisal and synthesis methods, results, limitations, conclusions and implications of key findings, systematic review registration number	P4
**Introduction**			
Rationale	3	Describe the rationale for the review in the context of what is already known	P7
Objectives	4	Provide an explicit statement of questions being addressed with reference to participants, interventions, comparisons, outcomes, and study design (PICOS)	P8
**Methods**			
Protocol and registration	5	Indicate if a review protocol exists, if and where it can be accessed (such as web address), and, if available, provide registration information including registration number	P9
Eligibility criteria	6	Specify study characteristics (such as PICOS, length of follow-up) and report characteristics (such as years considered, language, publication status) used as criteria for eligibility, giving rationale	P9
Information sources	7	Describe all information sources (such as databases with dates of coverage, contact with study authors to identify additional studies) in the search and date last searched	P10
Search	8	Present full electronic search strategy for at least one database, including any limits used, such that it could be repeated	P10
Study selection	9	State the process for selecting studies (that is, screening, eligibility, included in systematic review, and, if applicable, included in the meta-analysis)	P10
Data collection process	10	Describe method of data extraction from reports (such as piloted forms, independently, in duplicate) and any processes for obtaining and confirming data from investigators	P11
Data items	11	List and define all variables for which data were sought (such as PICOS, funding sources) and any assumptions and simplifications made	P11
Risk of bias in individual studies	12	Describe methods used for assessing risk of bias of individual studies (including specification of whether this was done at the study or outcome level), and how this information is to be used in any data synthesis	P12
Summary measures	13	State the principal summary measures (such as risk ratio, difference in means).	P12
Synthesis of results	14	Describe the methods of handling data and combining results of studies, if done, including measures of consistency (such as I^2 statistic) for each meta-analysis	P12
Risk of bias across studies	15	Specify any assessment of risk of bias that may affect the cumulative evidence (such as publication bias, selective reporting within studies)	Supplement 3
Additional analyses	16	Describe methods of additional analyses (such as sensitivity or subgroup analyses, meta-regression), if done, indicating which were pre-specified	-
**Results**			
Study selection	17	Give numbers of studies screened, assessed for eligibility, and included in the review, with reasons for exclusions at each stage, ideally with a flow diagram	P11
Study characteristics	18	For each study, present characteristics for which data were extracted (such as study size, PICOS, follow-up period) and provide the citations	Table 1 and Supplement 3
Risk of bias within studies	19	Present data on risk of bias of each study and, if available, any outcome-level assessment (see item 12).	P11

Results of individual studies	20	For all outcomes considered (benefits or harms), present for each study (a) simple summary data for each intervention group and (b) effect estimates and confidence intervals, ideally with a forest plot	P15
Synthesis of results	21	Present results of each meta-analysis done, including confidence intervals and measures of consistency	P15
Risk of bias across studies	22	Present results of any assessment of risk of bias across studies (see item 15)	P18
Additional analysis	23	Give results of additional analyses, if done (such as sensitivity or subgroup analyses, meta-regression) (see item 16)	-
**Discussion**			
Summary of evidence	24	Summarise the main findings including the strength of evidence for each main outcome; consider their relevance to key groups (such as health care providers, users, and policy makers)	P19
Limitations	25	Discuss limitations at study and outcome level (such as risk of bias), and at review level (such as incomplete retrieval of identified research, reporting bias)	P21
Conclusions	26	Provide a general interpretation of the results in the context of other evidence, and implications for future research	P22
**Funding**			
Funding	27	Describe sources of funding for the systematic review and other support (such as supply of data) and role of funders for the systematic review	P23

Information 2. Search Strategy

Pubmed

("embolectomy"[MeSH Terms] OR "embolectomy"[All Fields] OR "embolectomies"[All Fields] OR ("thrombectomy"[MeSH Terms] OR "thrombectomy"[All Fields] OR "thrombectomies"[All Fields]) OR "Endovascular"[All Fields]) AND ("stroke"[MeSH Terms] OR "stroke"[All Fields] OR "strokes"[All Fields] OR "stroke s"[All Fields] OR ("stroke"[MeSH Terms] OR "stroke"[All Fields] OR "cva"[All Fields]) OR ("stroke"[MeSH Terms] OR "stroke"[All Fields] OR ("cerebrovascular"[All Fields] AND "accident"[All Fields]) OR "cerebrovascular accident"[All Fields])) AND (("beyond"[All Fields] AND "24"[All Fields] AND "Hours"[All Fields]) OR ("Late"[All Fields] AND ("window"[All Fields] OR "window s"[All Fields] OR "windowed"[All Fields] OR "windowing"[All Fields] OR "windows"[All Fields])) OR ("Late"[All Fields] AND ("time"[MeSH Terms] OR "time"[All Fields])) OR ("Late"[All Fields] AND ("time"[MeSH Terms] OR "time"[All Fields])))

Cochrane Library

#1 MeSH descriptor: [Endovascular thrombectomy] explode all trees

#2 MeSH descriptor: [Stroke beyond 24 hours] explode all trees

#3 #1 AND #2

Embase

#1 'percutaneous thrombectomy'/exp OR 'percutaneous thrombectomy'

#2 'cerebrovascular accident'

#3 "beyond AND 24 AND hours"

#4 #1 AND #2 AND #3

Information 3 Assessment of risk of bias

Newcastle-Ottawa Scale quality assessment scale for cohort studies

Author, year	Representativeness of the exposed cohort	Selection of the nonexposed cohort	Ascertainment of exposure	Demonstration that outcome of interest was not present at start of study	Comparability of cohorts on the basis of the design or analysis	Assessment of outcome	Was follow-up long enough for outcomes to occur	Adequacy of follow up of cohorts	Score
Desai, 2018	✸	✸	✸	✸		✸	✸	✸	**7**
Dhillon, 2022	✸	✸	✸	✸	✸	✸		✸	**7**
Nguyen, 2023		✸	✸	✸	✸	✸	✸	✸	**7**
Purrucker, 2022	✸	✸	✸	✸			✸	✸	**6**
Ha, 2022		✸	✸	✸				✸	**4**
Shaban, 2022	✸	✸	✸	✸	✸		✸	✸	**7**
Wen, 2023		✸	✸	✸		✸	✸	✸	**6**

Overall score: 0-3 points = low quality; 4-6 points = intermediate quality; 7-9 points = high quality.

Information 4. Contour-enhanced meta-analysis funnel plots and Egger's test

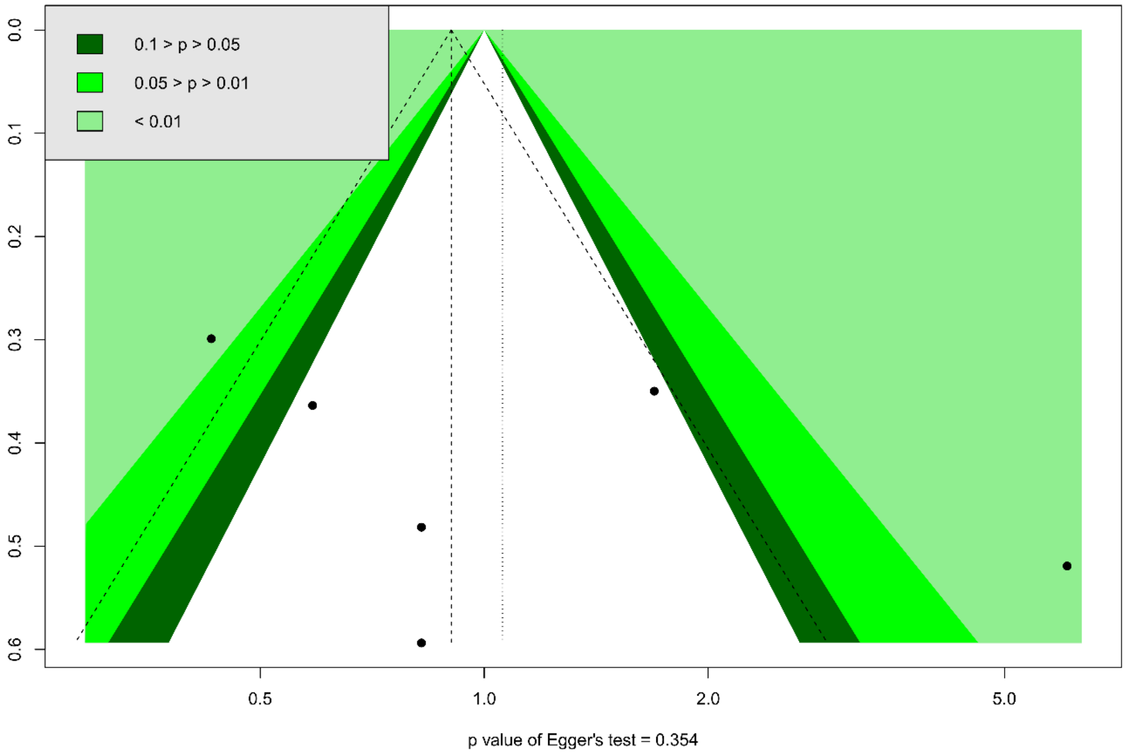

Contour-enhanced meta-analysis funnel plots: 90-day Modified Rankin Scale 0-2

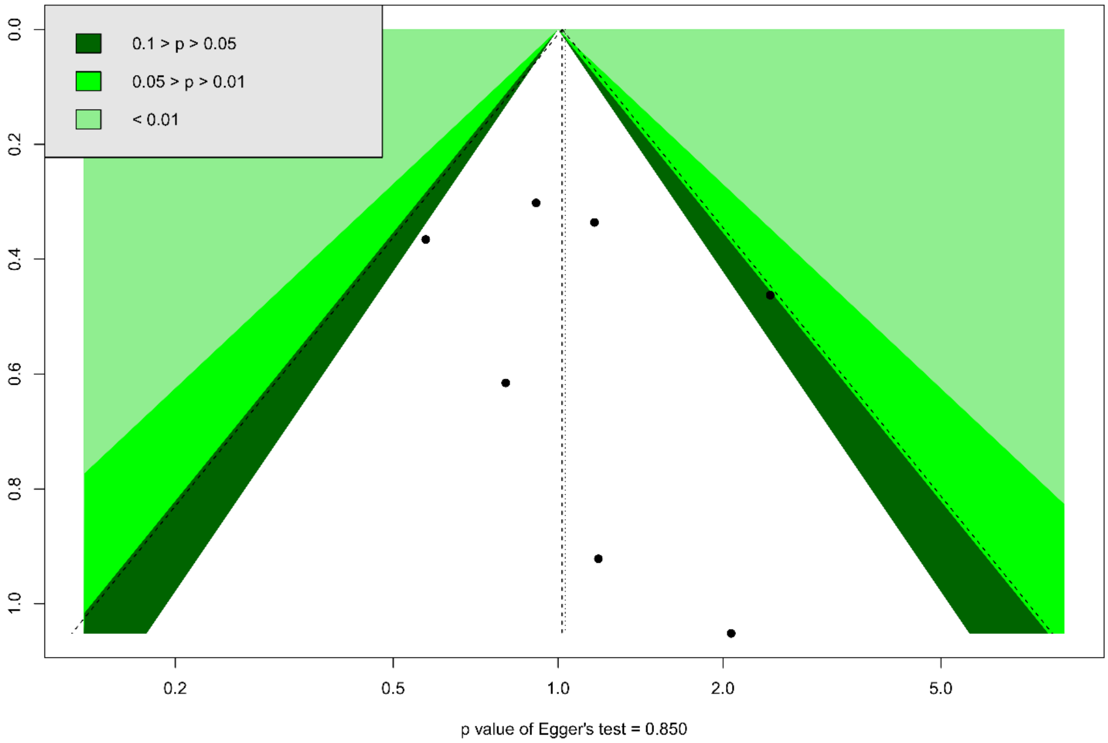

Contour-enhanced meta-analysis funnel plots: Thrombolysis in Cerebral Infarction score:2b-3

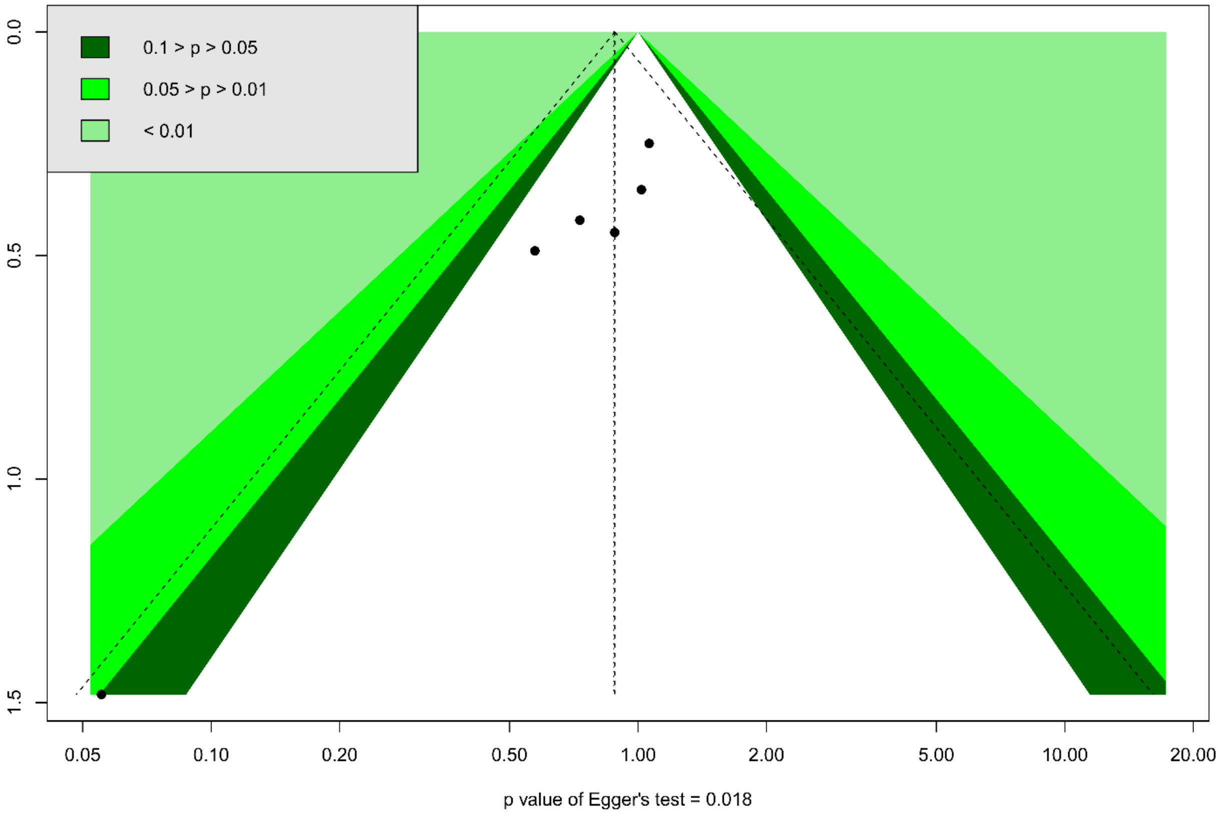

Contour-enhanced meta-analysis funnel plots: Any intracerebral hemorrhage

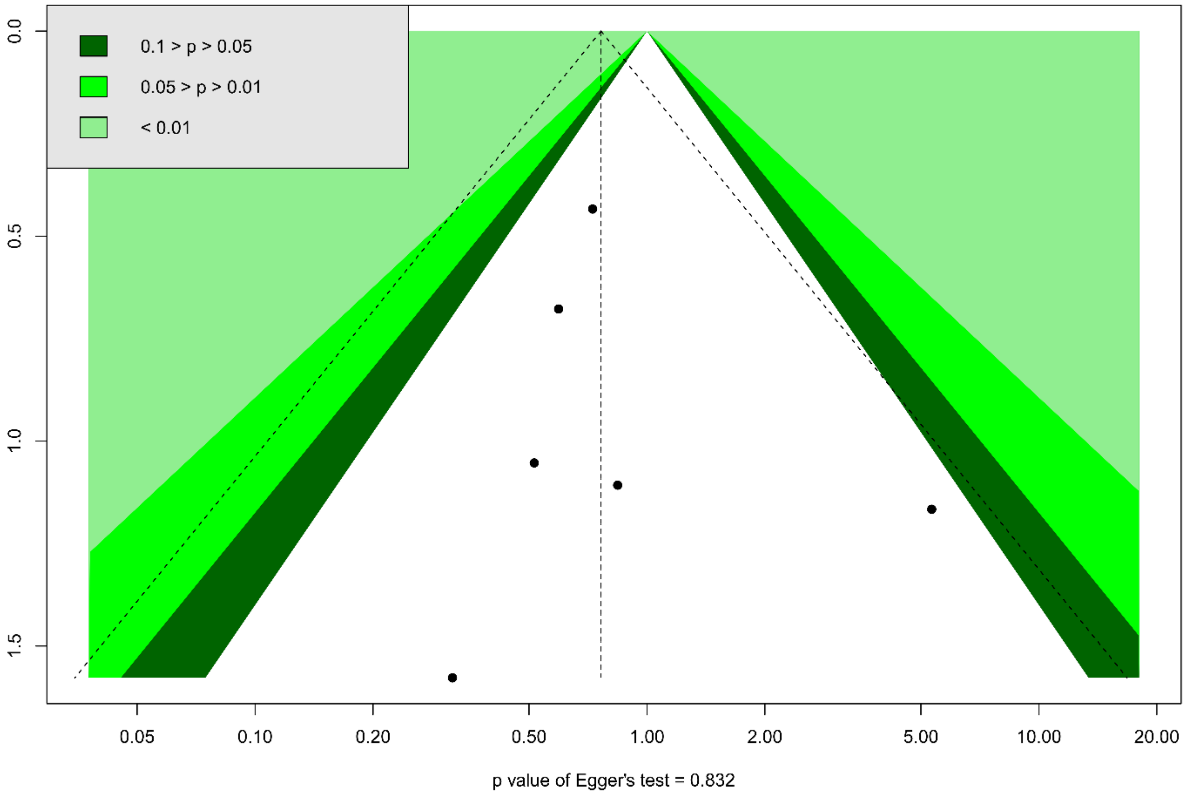

Contour-enhanced meta-analysis funnel plots: Symptomatic intracerebral hemorrhage

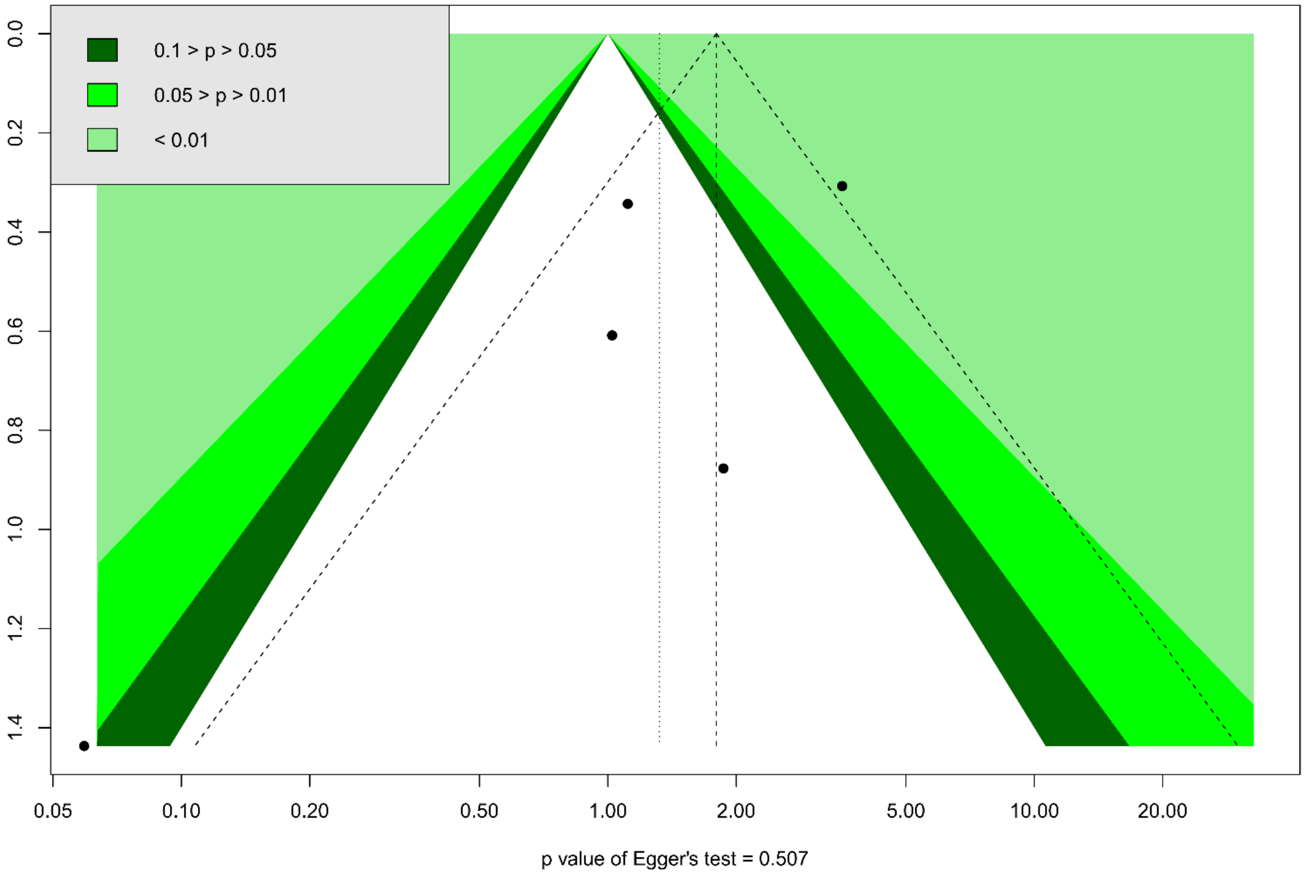

Contour-enhanced meta-analysis funnel plots: 90-day all-cause mortality

Information 5. Grading of Recommendations Assessment, Development, and Evaluation (GRADE)

EVT beyond 24 hr compared to EVT within 24 hr for patients with acute ischemic stroke with large vessel occlusion

Patient or population: patients with **acute ischemic stroke with large vessel occlusion**
Setting:
Intervention: Beyond 24 hr
Comparison: Within 24 hr

Certainty assessment							№ of patients		Effect		Certainty	Importance
№ of studies	Study design	Risk of bias	Inconsistency	Indirectness	Imprecision	Other considerations	[intervention]	[comparison]	Relative (95% CI)	Absolute (95% CI)		
**Good functional outcomes**												
6	non-randomised studies	serious[a]	very serious[b]	not serious	serious[c]	none	88/264 (33.3%)	1022/2929 (34.9%)	**OR 1.06** (0.51 to 2.19)	**13 more per 1,000** (from 134 fewer to 191 more)	⊕◯◯◯ Very low	CRITICAL
**Successful reperfusion**												
7	non-randomised studies	serious[a]	not serious	not serious	serious[c]	none	317/377 (84.1%)	2682/3156 (85.0%)	**OR 1.03** (0.72 to 1.48)	**4 more per 1,000** (from 47 fewer to 44 more)	⊕◯◯◯ Very low	CRITICAL
**Intracranial hemorrhage**												
6	non-randomised studies	serious[a]	not serious	not serious	serious[c]	publication bias strongly suspected[d]	74/322 (23.0%)	778/2992 (26.0%)	**OR 0.88** (0.64 to 1.21)	**24 fewer per 1,000** (from 76 fewer to 38 more)	⊕◯◯◯ Very low	CRITICAL
**Symptomatic intracranial hemorrhage**												
6	non-randomised studies	serious[a]	not serious	not serious	serious[c]	none	16/291 (5.5%)	56/787 (7.1%)	**OR 0.76** (0.41 to 1.40)	**16 fewer per 1,000** (from 41 fewer to 26 more)	⊕◯◯◯ Very low	CRITICAL
**90-day all-cause mortality**												
5	non-randomised studies	serious[a]	not serious	not serious	serious[c]	none	49/167 (29.3%)	706/2825 (25.0%)	**OR 1.32** (0.55 to 3.19)	**56 more per 1,000** (from 95 fewer to 265 more)	⊕◯◯◯ Very low	CRITICAL

CI: confidence interval; **OR:** odds ratio

Explanations

a. Only the studies conducted by Nguyen and Shaban are of high quality; the remaining studies are of intermediate quality.
b. There is substantial heterogeneity, and some variability in the point estimates in the forest plots.
c. The 95% CI included a odds ratio of 1.0, and the total number of events or patients did not exceed the required information size.
d. The small number of studies analyzed for this specific outcome in our meta-analysis may affect the evaluation of publication bias.

Information 6: Scatter plot association between time from Last Known Well (TLKW) to groin puncture and pooled odds ratios (ORs) for five clinical outcomes

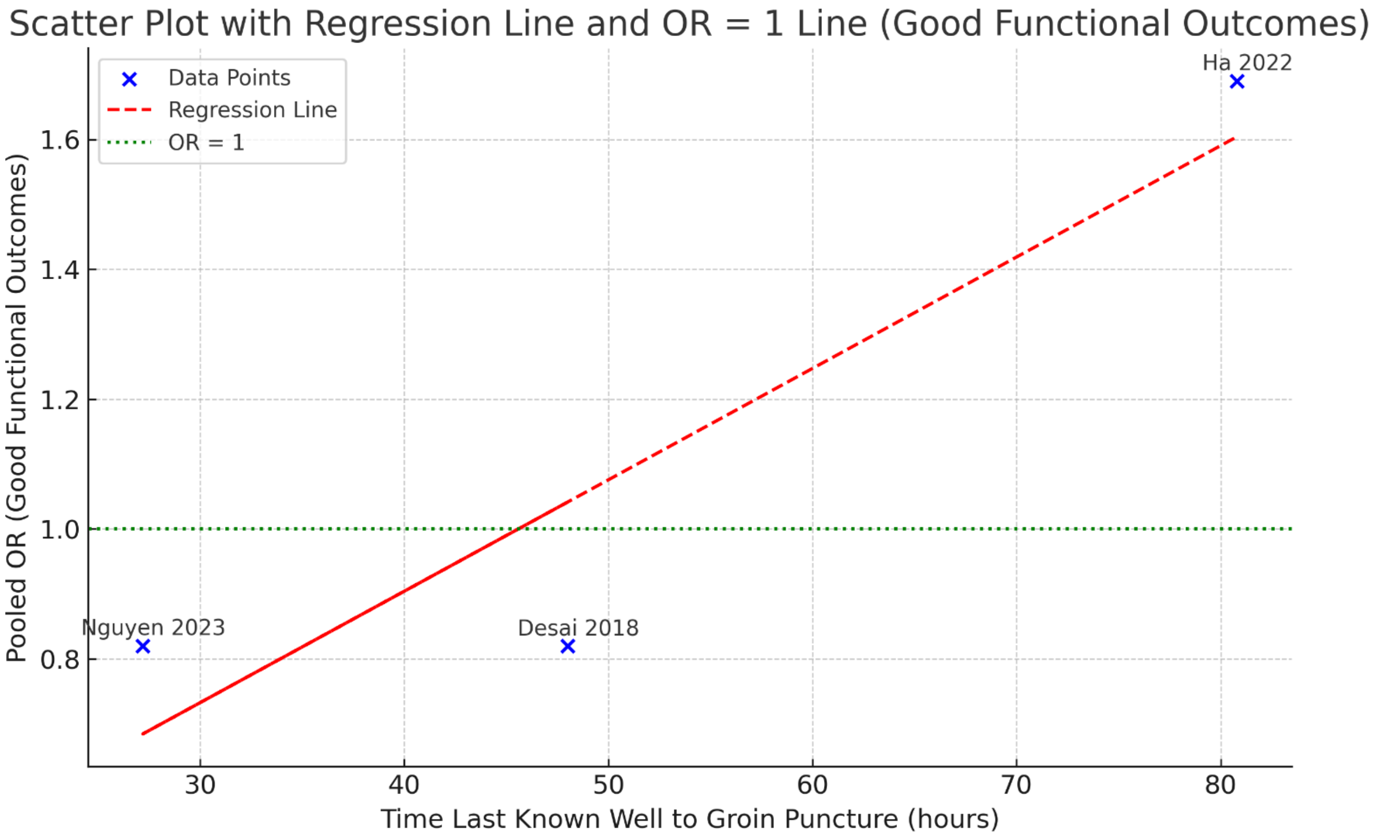

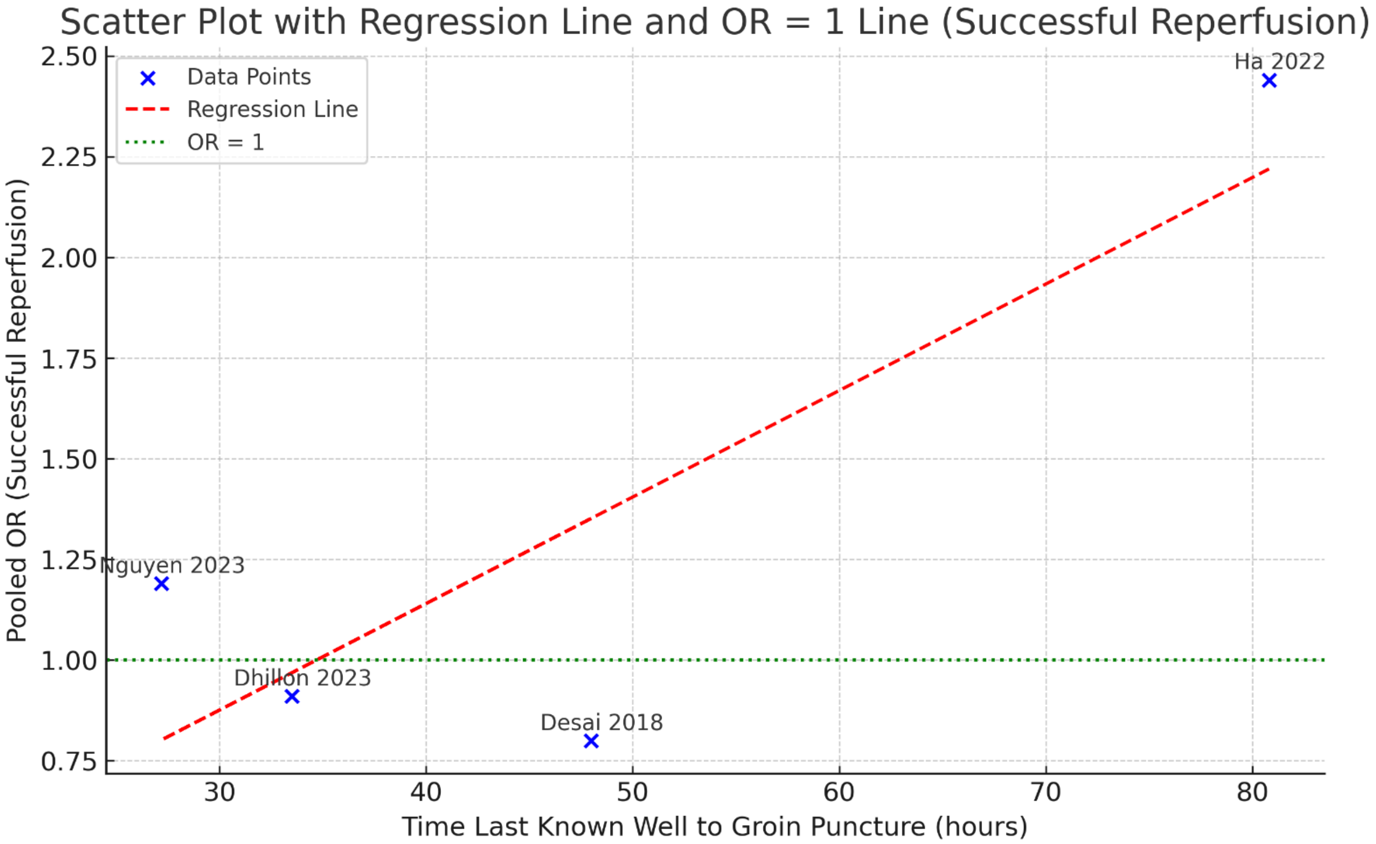

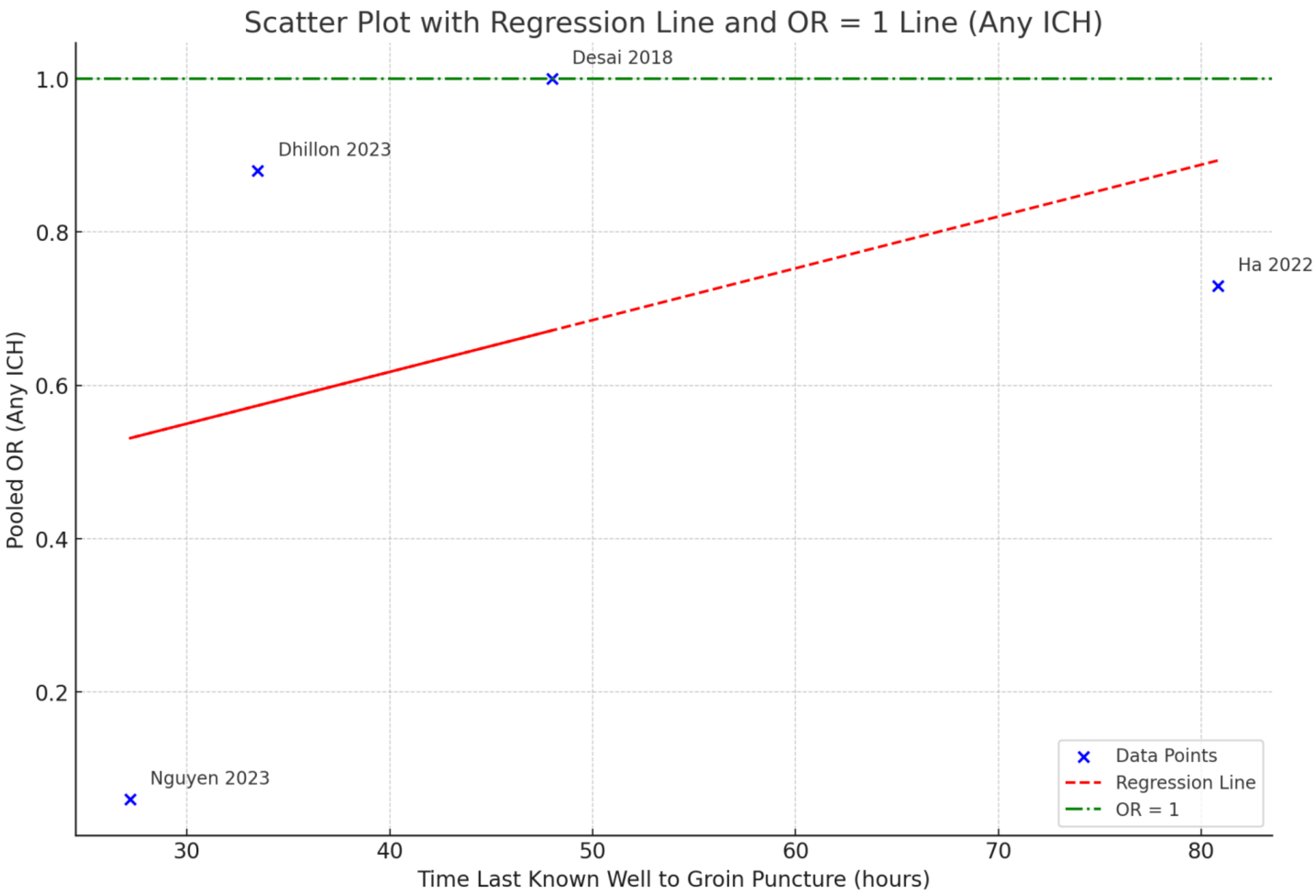
Scatter Plot with Regression Line and OR = 1 Line (Any ICH)
Desai 2018
Dhillon 2023
Ha 2022
Nguyen 2023
Data Points
Regression Line
OR = 1
Pooled OR (Any ICH)
Time Last Known Well to Groin Puncture (hours)
1.0
0.8
0.6
0.4
0.2
30
40
50
60
70
80

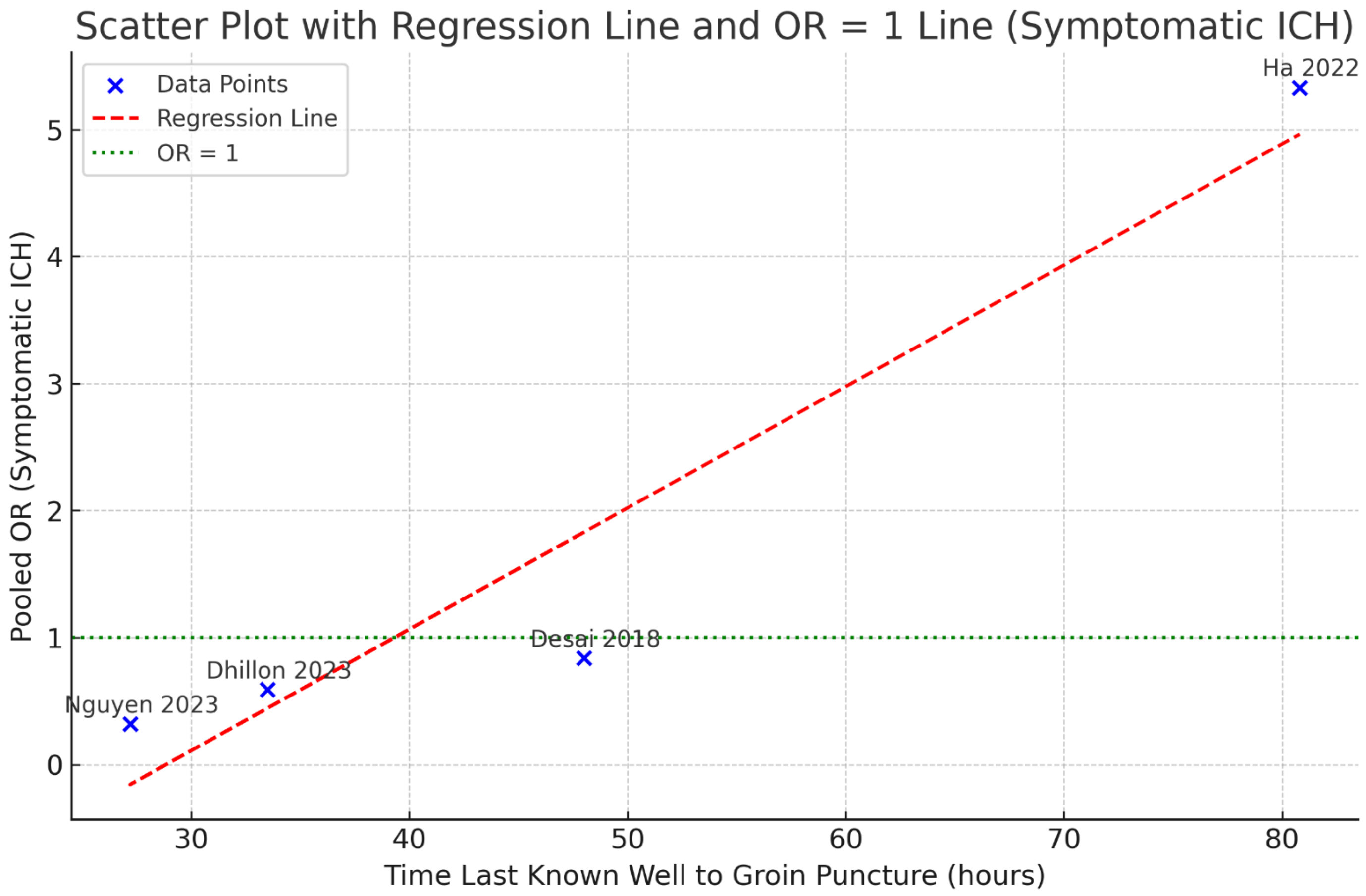
Scatter Plot with Regression Line and OR = 1 Line (Symptomatic ICH)
Data Points
Regression Line
OR = 1
Ha 2022
Desai 2018
Dhillon 2023
Nguyen 2023
Pooled OR (Symptomatic ICH)
Time Last Known Well to Groin Puncture (hours)
5
4
3
2
1
0
30
40
50
60
70
80

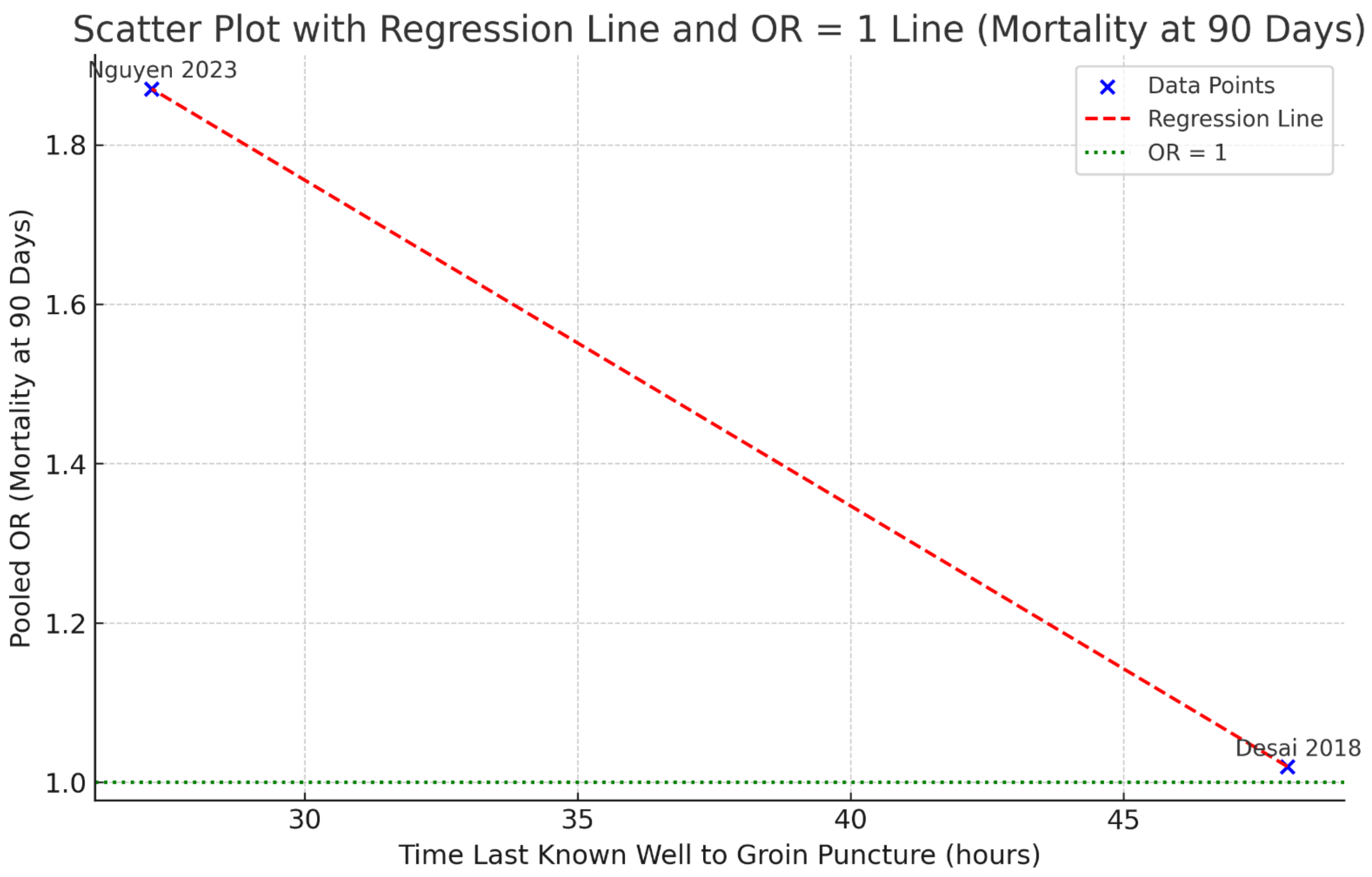
Scatter Plot with Regression Line and OR = 1 Line (Mortality at 90 Days)
Nguyen 2023
Desai 2018
Data Points
Regression Line
OR = 1
Pooled OR (Mortality at 90 Days)
1.8
1.6
1.4
1.2
1.0
30
35
40
45
Time Last Known Well to Groin Puncture (hours)

Information 7. Detail of study characteristics

Desai, J 2018

Methods	Retrospectively, a multi-center cohort study
Participants	1. 128 patients (>24hr: 21, 6-24hr: 107)<br>2. Location: Unite State<br>3. Mean age: 68.7 years old, male: 39.1%
Inclusion criteria	1. Acute ischemic stroke treated with EVT beyond 24 hours since TLKW (including wake up, witnessed and unwitnessed strokes)<br>2. Presence of age-based mismatch between clinical severity of stroke and infarct core<br>3. Image: Volume by CT perfusion or DWI-MRI using automated software RAPID (iSchemaView, Menlo Park, California, USA).<br>A. Age <80 years: NIHSS score ≥10, infarct core volume <31 mL or NIHSS score ≥20 and infarct core volume <51 mL<br>B. Age ≥80 years: NIHSS score ≥10 mL and infarct core volume <21 mL
Occlusion location	1. Intracranial internal carotid artery (ICA)<br>2. First segment of middle cerebral artery (MCA M1)
Exclusion criteria	1. More than one-third of the MCA territory involvement<br>2. Hemorrhage on head CT
Intervention	1. Intervention: EVT beyond 24 hours since TLKW<br>2. Comparison: EVT between 6-24 hours since TLKW<br>2. Duration of follow-up (post-treatment to study end): 90-day
Outcome	1. Procedure outcome: Rates of TICI ≥2b<br>2. Efficacy outcomes: Early neurological recovery、mRS 0–2 at 90 days<br>3. Safety outcomes: Neurologic deterioration、Symptomatic ICH、Mortality
Symptomatic ICH definition	European Acute Stroke Study III criteria: the presence of extravascular blood in the cranium that was associated with an increase in the NIHSS score of ≥4 points or death) within 24 hours after thrombectomy.
Note	NIHSS: National Institutes of Health Stroke Scale, EVT: Endovascular thrombectomy<br>TLKW: Time last known well, mTICI: modified Thrombolysis in Cerebral Infarction<br>mRS: Modified Rankin Scale

Dhillon, J 2022

Methods	Prospectively, a propensity score matched cohort study
Participants	1. 1150 patients (>24hr: 104, 6-24hr: 1046), After PSM:312 (>24hr: 104, 6-24hr: 208)<br>2. Location: England<br>3. After PSM Male: 60.3%
Inclusion criteria	1. Acute ischemic stroke who received EVT<br>2. The selection of EVT eligible patients was at the discretion of the practitioners based on each institution's protocol.<br>3. Image: CT angiography and/or perfusion-based imaging, parenchymal imaging findings and clot location were not available.
Occlusion location	1. No specific limits were applied to the clinical inclusion criteria.
Exclusion criteria	1. Patients with missing discharge mRS<br>2. Acute ischemic stroke presenting within 6 hours
Intervention	1. Intervention: EVT beyond 24 hours since TLKW<br>2. Comparison: EVT between 6-24 hours since TLKW<br>3. Duration of follow-up (post-treatment to study end): 6-month
Outcome	1. Primary outcome: mRS score at hospital discharge and 6 months<br>2. Functional outcome: Early neurological deterioration and early neurological Improvement in 24 hours、mTICI ≥2b<br>3. Safety outcome:、Any ICH、Symptomatic ICH、Inhospital mortality
Symptomatic ICH definition	European Collaborative Acute Stroke Study (ECASS) II: any ICH with an increase in NIHSS score of ≥4 within 24 hours or death.
Note	NIHSS: National Institutes of Health Stroke Scale, EVT: Endovascular thrombectomy<br>mTICI: modified Thrombolysis in Cerebral Infarction, mRS: Modified Rankin Scale<br>TLKW: Time last known well

Nguyen 2023

Methods	Retrospectively, a propensity score matched cohort study
Participants	1. 166 patients (>24hr: 20, 6-24hr: 146), After PSM: 49 (>24hr: 18, 6-24hr: 31)<br>2. Location: Vietnam<br>3. After PSM Mean age: 59.4 years old, male: 81.6 %
Inclusion criteria	1. Acute ischemic stroke more than 6 h since TLKW<br>2. Age ⩾18 years;<br>3. mRS score of 0–2<br>4. Met DAWN or DEFUSE 3 criteria<br>5. Image: CT or MR angiography evidence of LVO, using The RAPID AI (iSchemaView, Menlo Park, CA, USA)
Occlusion location	1. Intracranial internal carotid artery (ICA)<br>2. First segment of middle cerebral artery (MCA M1)<br>3. Proximal M2 segments of middle cerebral artery (Proximal MCA M2)<br>4. Tandem occlusions
Exclusion criteria	1. No mention
Intervention	1. Intervention: EVT beyond 24 hours since TLKW<br>2. Comparison: EVT between 6-24 hours since TLKW<br>3. Duration of follow-up (post-treatment to study end): 90-day
Outcome	1. Primary outcome: mRS 0–2 at 90 days<br>2. Secondary outcomes: mRS ordinal shift、mTICI ≥2b<br>3. Safety outcomes: ICH、Symptomatic ICH、Mortality
Symptomatic ICH definition	The SITS-MOST definition : A local or remote type II parenchymal haemorrhage within 22 to 36 hours after treatment (or sooner) associated with a ≥ fourpoint deterioration on the NIHSS score from baseline or from the lowest score from baseline to 24 hours, or leading to death.
Note	NIHSS: National Institutes of Health Stroke Scale, EVT: Endovascular thrombectomy<br>TLKW: Time last known well, mTICI: modified Thrombolysis in Cerebral Infarction<br>mRS: Modified Rankin Scale, CTA: computed tomographic angiography, MRA (magnetic resonance angiography), TLKW: Time last known well, PSM: Propensity score matched

Purrucker 2022

Methods	Two local prospective recanalization database cohort study
Participants	1. 2347 patients (>24hr: 43, 6-24hr: 2304)<br>2. Location: German<br>3. Mean age: 73.9 years old, male: 48.2 %
Inclusion criteria	1. If LVO was present and EVT had been started.<br>2. The decision to perform EVT was left to the discretion of the treating physicians.<br>a. Patients presenting with supratentorial ischemia, NIHSS ⩾ 6<br>3. Image: CT or MR angiography evidence of LVO.
Occlusion location	1. Intracranial internal carotid artery (ICA)<br>2. First segment of middle cerebral artery (MCA M1)<br>3. Proximal M2 segments of middle cerebral artery (Proximal MCA M2)<br>4. Tandem occlusions
Exclusion criteria	1. No mention.
Intervention	1. Intervention: EVT beyond 24 hours since TLKW<br>2. Comparison: EVT between 9-24 hours since TLKW<br>3. Duration of follow-up (post-treatment to study end): 90-day
Outcome	1. Functional outcome: mRS at 90 days、ICH、sICH<br>2. Radiological outcome: mTICI≥2b
Symptomatic ICH definition	The SITS-MOST definition : A local or remote type II parenchymal haemorrhage within 22 to 36 hours after treatment (or sooner) associated with a ≥ fourpoint deterioration on the NIHSS score from baseline or from the lowest score from baseline to 24 hours, or leading to death.
Note	NIHSS: National Institutes of Health Stroke Scale, EVT: Endovascular thrombectomy<br>TLKW: Time last known well, mTICI: modified Thrombolysis in Cerebral Infarction<br>mRS: Modified Rankin Scale, CTA: computed tomographic angiography, MRA (magnetic resonance angiography), TLKW: Time last known well, PSM: Propensity score matched. LVO: Large vessel occlusion

SH Ha 2022

Methods	Retrospective, two center cohort study
Participants	1. 165 patients (>24hr: 61, 6-24hr: 104)<br>2. Location: German<br>3. Mean age: 73.9 years old, male: 48.2 %
Inclusion criteria	1. If LVO was present and EVT had been started.<br>2. The decision to perform EVT was left to the discretion of the treating physicians.<br>a. Patients presenting with supratentorial ischemia, NIHSS ⩾ 6<br>3. Image: MRI with a 3.0T Philips scanner (Philips Healthcare, Eindhoven, The Netherlands)<br>A. Olea Sphere® imaging system (Olea Medical SAS,La Ciotat, France) for automatic postprocessing of perfusionweighted imaging (PWI) and DWI studies.<br>B. The volume of hypoperfused brain tissue was calculated with time to maximum perfusion (defined as >6 s). Based on these parameters, the ratio of PWI to DWI was also measured to define core-penumbra mismatch.
Occlusion location	1. Intracranial internal carotid artery (ICA)<br>2. First segment of middle cerebral artery (MCA M1)<br>3. Proximal M2 segments of middle cerebral artery (Proximal MCA M2)<br>4. Tandem occlusions
Exclusion criteria	1. No mention.
Intervention	1. Intervention: EVT beyond 24 hours since TLKW<br>2. Comparison: EVT between 6-24 hours since TLKW<br>3. Duration of follow-up (post-treatment to study end): 90-day
Outcome	1. Functional outcome: mRS at 90 days、ICH、sICH<br>2. Radiological outcome: mTICI≥2b
Symptomatic ICH definition	The SITS-MOST definition : A local or remote type II parenchymal haemorrhage within 22 to 36 hours after treatment (or sooner) associated with a ≥ fourpoint deterioration on the NIHSS score from baseline or from the lowest score from baseline to 24 hours, or leading to death.
Note	NIHSS: National Institutes of Health Stroke Scale, EVT: Endovascular thrombectomy<br>TLKW: Time last known well, mTICI: modified Thrombolysis in Cerebral Infarction<br>mRS: Modified Rankin Scale, CTA: computed tomographic angiography, MRA (magnetic resonance angiography), TLKW: Time last known well, LVO: Large vessel occlusion

Shaban 2022

Methods	Retrospective, multicenter, a propensity score matched cohort study
Participants	1. 1942 patients (>24hr: 121, 6-24hr: 1821), After PSM: 305 patients (>24hr: 91, 6-24hr: 214)<br>2. Location: USA, France, Germany, Switzerland, South Korea, Uruguay, and Argentina<br>3. Mean age: 68.0 years old, male: 49.6 %
Inclusion criteria	1. All patients who underwent MT for large vessel occlusion.<br>2. Image: not mention
Occlusion location	1. Anterior circulation and posterior circulation occlusions were included
Exclusion criteria	1. No mention.
Intervention	1. Intervention: EVT beyond 24 hours since TLKW<br>2. Comparison: EVT between 6-24 hours since TLKW<br>3. Duration of follow-up (post-treatment to study end): 90-day
Outcome	1. Primary outcome: functional independence (mRS 0-2 at 90 days)、ICH、sICH<br>2. Primary safety outcome: sICH、mortality at 90 days<br>3. Secondary outcomes: Successful recanalization (mTICI≥2b)、factors associated with good outcomes in patients undergoing thrombectomy beyond 24 hours
Symptomatic ICH definition	Defined as an increase in NIHSS by 4 points in 24 hours.
Note	NIHSS: National Institutes of Health Stroke Scale, EVT: Endovascular thrombectomy<br>TLKW: Time last known well, mTICI: modified Thrombolysis in Cerebral Infarction<br>mRS: Modified Rankin Scale, CTA: computed tomographic angiography, MRA (magnetic resonance angiography), TLKW: Time last known well, PSM: Propensity score matched, LVO: Large vessel occlusion

Wen 2023

Methods	Prospective, single center cohort study
Participants	1. 239 patients (>24hr: 25, 6-24hr: 214)<br>2. Location: China<br>3. Mean age: 67.3 years old, male: 61.9 %
Inclusion criteria	1. The patient with acute ischemic stroke due to anterior circulation large-vessel occlusion or stenosis<br>2. 18 years or older<br>3. mRS score ≤ 2 before AIS onset<br>4. NIHSS scores ≥6 before EVT<br>5. Image: CT or MR angiography evaluated the occluded or stenotic vessels<br>A. EVT was considered if the infarcted area did not cover more than one-third of the MCA blood supply territory and if there were patients with a penumbral region, at the joint discretion of the neurology and neurointerventional physicians
Occlusion location	1. Anterior circulation Involving ICA, MCA M1, or MCA M2
Exclusion criteria	1. Data were excluded if the clinical information (time since symptom onset or last seen well, treatment with EVT, etc.) was incomplete or the patients declined to participate in the follow-up
Intervention	1. Intervention: EVT beyond 24 hours since TLKW<br>2. Comparison: EVT between 6-24 hours since TLKW<br>3. Duration of follow-up (post-treatment to study end): 90-day
Outcome	1. Efficacy outcome: mTICI≥2b、mRS at 90 days<br>2. Safety outcome: ICH、sICH、mortality at 90 days
Symptomatic ICH definition	Defined as imaging-confirmed ICH on imaging by NIHSS scores of ≥4 within 7 days of EVT
Note	NIHSS: National Institutes of Health Stroke Scale, EVT: Endovascular thrombectomy<br>TLKW: Time last known well, mTICI: modified Thrombolysis in Cerebral Infarction<br>mRS: Modified Rankin Scale, CTA: computed tomographic angiography, MRA (magnetic resonance angiography), TLKW: Time last known well, LVO: Large vessel occlusion
